# Echocardiographic Assessment of Prosthetic Valves

**DOI:** 10.31083/j.rcm2310343

**Published:** 2022-10-11

**Authors:** Hasan Ashraf, William K Freeman

**Affiliations:** ^1^Department of Cardiology, Yale University School of Medicine, New Haven, CT 06510, USA; ^2^Department of Cardiovascular Disease, Division of Echocardiography, Mayo Clinic, Scottsdale, AZ 85259, USA

**Keywords:** prosthetic cardiac valves, echocardiography, Doppler, mechanical valve, bioprosthetic valve, structural valve degeneration, prosthetic valve thrombosis

## Abstract

Prosthetic valves are increasingly encountered in clinical practice. A grasp of the intricacies of the assessment and management of prosthetic valves is thus a crucial skillset for the practicing cardiologist. Echocardiography is the imaging modality of choice for the anatomic and functional evaluation of prosthetic valve. This document reviews the general features of prosthetic valves, echocardiographic identification of normally functioning and dysfunctional prosthetic valves as well as echocardiographic diagnosis of specific prosthetic valvular abnormalities.

## 1. Introduction

Valvular heart disease is an increasingly prevalent global problem and is expected to only grow with the rising age of the world’s population [1]. Advances in replacement of diseased heart valves through standard surgical or transcatheter prosthetic valve implantation have revolutionized the management of valvular heart disease and have allowed for increasing number of patients that can be treated and with significantly fewer complications than before [2, 3, 4]. Prosthetic valves have also been demonstrated to decrease mortality and improve quality of life [5, 6, 7]. Nevertheless, because of the substitution of native valve with a foreign body, prosthetic valve implantation is concomitant with a host of complications, some of which may be expected given the natural history of the prosthetic valve. As a result, patients require lifelong monitoring which can be performed with a number of modalities that provide anatomic and functional information of the prosthesis. Transthoracic echocardiography (TTE) is ideal for this purpose, as it provides a rapid noninvasive modality for the assessment of prosthetic valve structure and function both immediately after prosthetic implantation and during long-term follow-up. TTE has thereby become the mainstay in the diagnosis and management of prosthetic valve disease.

Despite the widespread availability of echocardiography, assessment of prosthetic valve structure and function is more technically challenging than that of native valves. Even assessment of a normally functioning prosthesis may not be straightforward because of acoustic shadowing and artifacts, and therefore requires a combination of 2-Dimensional imaging as well as Doppler echocardiography to come to a correct conclusion. This review will provide an overview of the echocardiographic assessment of prosthetic valves, including general principles that should direct the interpretation of a prosthetic valve study. Focus will be given to aortic and mitral prostheses, as they are encountered more frequently in clinical practice. Additionally echocardiographic determination and differentiation of complications will be reviewed.

## 2. A Systematic Approach to Prosthetic Valves

A consistent and methodological approach should be undertaken with all prostheses, regardless of the location or the type of prosthesis. This ensures that critical information is not overlooked, allows for identification of any change in prosthetic valve function, and the detection of any prosthetic valve complications.

Prior to echocardiographic imaging, the patient’s chart and operative notes should be reviewed to determine the age, location, type, and size of the prosthesis. Additional procedures performed during the index operation may be pertinent for accurate echocardiographic interpretation, such as an aortic root surgery. It is also worthwhile to review intraoperative transesophageal echocardiographic images and post-operative echo images to compare with current imaging. When such information is not readily available in the medical record, it is often the case that the patient carries a medical card that allows for the identification of some of the above information.

At the time of the echocardiographic study, routine vitals including blood pressure and heart rate should be taken. The heart rate is particularly critical for Doppler assessment of a mitral valve prosthesis (MVP), as the gradient is dependent on the diastolic filling time. Additionally, the patient body surface area should be reviewed, given the impact it has on optimal prosthesis size and the possibility of pathological states with undersized prostheses leading to patient-prosthesis mismatch (PPM).

A standard and complete echocardiographic evaluation of prosthetic valves will include 2-dimensional (2-D) images that are obtained from multiple angles of interrogation and may require off-axis and non-standard views. In some situations where significant technical difficulty in imaging the prosthesis is encountered through standard TTE, transesophageal echocardiography (TEE) may be necessary, particularly in the mitral position. Nevertheless, despite this, many complications of prosthetic valves can be identified by 2-D TTE before even hemodynamic assessment using Doppler, such as valve thrombosis, pannus formation, and endocarditis. Regardless of the type of the prosthesis, close attention should be paid to the seating of the prosthesis, the interface of the sewing ring and annulus, and the motion and degree of opening of the leaflets or occluders. The general stability of the prosthesis should be assessed, as movement of the prosthesis, typically in a rocking motion, may signify prosthetic dehiscence. Additionally, attention should be directed to any echo density present on the prosthesis, whether the occluder or the leaflet(s), but also on the cage, struts, or the sewing ring itself, as this may signify the thrombus or vegetation. In addition to the valve itself, standard assessment of the cardiac chamber sizes, assessment of ventricular systolic and diastolic function, and ventricular wall thickness should always be performed to determine the effect of any valvular disease on the rest of the myocardium.

Doppler echocardiography is an essential complement to 2-D imaging. The general principles and physics of pulsed wave (PW), continuous wave (CW), and color doppler are the same as those that are used for the assessment of native valves. This includes interrogation of the prosthetic valve from a number of angulations to permit optimal parallel alignment of the Doppler beam with blood flow. This may require non-standard interrogations of the valve; for instance, the highest velocity of the aortic prosthesis is most commonly obtained from the right parasternal window in elderly patients because the anterior movement of the cardiac chambers with aging results in more of an acute angle between the aortic root and the ventricular septum [8]. Color Doppler will also identify valvular regurgitation, and the anatomy of the regurgitation (intravalvular vs paravalvular). It can also demonstrate stenosis of a prosthesis by exhibiting significant turbulence of blood flow through the stenotic orifice.

Spectral Doppler is essential in yielding a number of hemodynamic parameters characterizing the prosthesis, such as mean and peak velocities and gradient, effective orifice area (EOA) and others. Some parameters are obtained in all prostheses, regardless of location. Other parameters, such as pressure halftime, dimensionless index (DVI), and acceleration time are only obtained depending on the anatomic location of the prosthetic valve. The details regarding these parameters, and their interpretation, will be further elaborated in subsequent sections of this review. Regardless, no one parameter may be used to make a diagnosis, and the collective data integrating the full 2-D imaging and doppler parameters should be used to arrive at the correct determination of the valve function.

As always, studies should be compared with any prior imaging if available, with any changes noted on the final report.

## 3. Echocardiographic Assessment: General Features of Prosthetic Valves

The type and design of the prosthesis will play a significant role in its echocardiographic assessment, as there is a significant amount of variation that characterizes the fluid dynamics for each design.

**Fig. 1.**
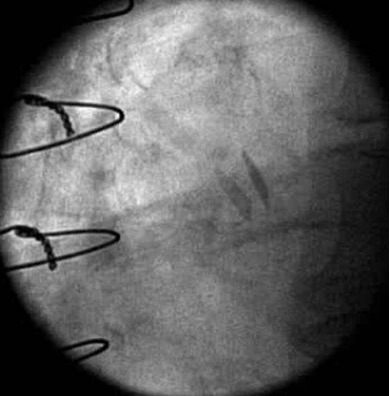
**Maximal opening of a 21 mm SJM Regent Mechanical Heart Valve in the aortic position with normal valve function**. Fluoroscopy demonstrated brisk and complete opening and closing of the leaflets. Note the leaflets are not quite 90 degrees perpendicular to the annular plane at the time of maximal opening which still is within normal limits.

Mechanical valves have three basic types that have historically been used in clinical practice, and even without prior knowledge regarding the mechanical valve type, 2-D echo can usually lead to accurate identification of the prosthesis type. The bileaflet valve SJM Regent Mechanical Heart Valve (acquired by Abbott, Santa Clara, CA, USA) is the most commonly implanted mechanical prosthesis in the world [9, 10]. These valves consist of two semicircular disks with a narrow orifice along the center between the two disks and two larger lateral semicircular orifices. The disks open 75–90 degrees relative to the annular plane, and are easily identified with 2-D echo given the significant acoustic shadowing that results (Fig. 1). However, the degree of disc motion and opening is not always identifiable by 2-D echo. The degree of opening of bileaflet prostheses is better evaluated in the mitral position, as it can be identified in 77% and 100% of patients with TTE and TEE respectively. This drops to 13 and 35% respectively in the aortic position [11]. This has substantial significance to the specificity of 2-D echo in identifying complete opening of a leaflet prosthesis. The motion should be brisk and essentially consistent with each beat, though there may be intermittent changes in transprosthetic gradients that lead to variation in the degree of opening, and therefore conclusions should be drawn only after examination of several consecutive beats.

In recent years, the newer generation On-X bileaflet valve has increasingly been implanted, most commonly in the aortic position. Its improved structural material devoid of silicon and improved engineering have led to improved fluid dynamics and reduced thrombogenicity [12, 13]. Echocardiographically, however, the On-X would essentially appear similar to a standard SJM Regent valve, as their structural differences are not substantial enough to allow for visual differentiation.

Other types of mechanical valves include the tilting disk valve (or monoleaflet valve) which utilizes a single disk that is circular in shape, and which rotates 70–75 degrees within the annulus. As a result, the cross-sectional area of the major orifice is semicircular when the disk is maximally opened; a consequence of the non-perpendicular position of the disk is that gradients across single disk valves are increased when compared to bileaflet valves [14, 15]. A third type of mechanical valve is the Starr-Edwards ball in cage valve, which is no longer routinely implanted because of its high thrombogenicity and unfavorable hemodynamics. Nevertheless, it was the first commercially available prosthetic valve and has historically been very durable, and may therefore still be encountered in clinical practice. Two-dimensional echo identification of this prosthesis is quite straightforward, as there will be obvious silicon ball movement into and out of the cage throughout the cardiac cycle. Of note, because the velocity of ultrasound in the silastic ball is slowed, propagation speed error artifact may ensue due to assumption of the standard speed of ultrasound in tissue. This results in the depiction of the ball as ovaloid rather than round and as an expected distortion with the ball in cage prosthesis, should not be interpreted as pathological [16].

Biological prostheses typically are stented or stentless xenografts, though homograft valves composed of cryopreserved human aortic or pulmonary valves are also commercially available. Traditionally they compose of three leaflets composed of a porcine aortic valve or bovine pericardium. The anatomy of bioprostheses, therefore resembles that of a native aortic valve. The theoretical benefit of stentless valves is their increase in EOA as well as the decreased stress on the cusp leading to improved durability and decreased risk of thrombosis [14, 17, 18, 19, 20]. Stentless bioprostheses are customarily limited to the aortic position [21].

Other less common prosthetics include homografts and autografts; the former are harvested using cadaveric aortic valves and implanted in the aortic root position via a total root replacement. The latter, which is implanted employing the Ross procedure, involves an alternative to aortic valve replacement with a mechanical or bioprosthetic valve whereby the aortic valve is replaced with a pulmonic autograft. In recent years, percutaneous valvular replacement techniques have dramatically improved, and transcatheter bioprostheses are more commonly encountered in clinical practice. The vast majority of transcatheter valves are implanted in the aortic position. The echocardiographic interpretation of these valves is quite similar to that of conventional prosthetic valves with only minor differences. As such these will not be dealt with separately in this review.

Blood flow through a normally functioning prosthetic valve will differ greatly from that through a native valve. The specific pattern of antegrade flow is specific to the valve and will vary based on the morphology of the valve and its number of orifices. It should be noted that a certain degree of stenosis is inherently present across all mechanical and bioprosthetic valves, and therefore a normally functioning prosthetic valve will exhibit similar hemodynamics with Doppler echocardiography to those of a mildly stenotic native valve [22]. The inherent stenosis is magnified as the prosthetic valve size becomes smaller. Conversely, a minimal amount of regurgitation also characterizes a normally functioning prosthetic valve, whether mechanical or even at times, bioprosthetic. This regurgitation may be seen on color Doppler with closure of the prosthesis occluders, leading to displacement of blood, or may be true regurgitation occurring at the hinges of the occluders. This latter trivial or mild regurgitant volume serves to maintain dynamic flow across the valve as a “washing jet”, and thereby reduce the risk of prosthetic thrombosis, particularly in the case of mechanical prostheses where the risk is appreciably greater. Bioprosthetic valves may also present with a trivial degree of regurgitation, typically identified in 10% of normally functioning bioprostheses [9].

Comparison of the size and subsequent hemodynamic profiles of the various prostheses is rendered challenging because of nonuniformity in sizing convention among different manufacturers [23]. A valve’s hemodynamic profile is predominantly determined by its internal diameter, and for a given labeled size, valves have a significant distribution of actual internal and external diameters [24]. The hemodynamic profiles of the range of prosthetic valves that are used or have been used in clinical practice is readily available [22]. A common error that may be encountered when the prosthesis size as listed by manufacturer labeling is not available prior to echocardiographic interpretation is the assumption that the prosthesis size is equal to the left ventricular outflow tract (LVOT) diameter. In fact, equating these two parameters may lead to gross overestimation of the true EOA by as much as 15–20%. Apart from the issue of internal and external diameters, even computing an EOA using the internal diameter is not reflective of the true EOA as the EOA is a functional area of blood flow, which is smaller than the internal surface area theoretically available for fluid flow in the valve.

## 4. Echocardiographic Assessment: Complications of Prosthetic Valves

Patients with valvular heart disease bear a high burden of morbidity and mortality, and even with intervention in the form of prosthetic valves, overall survival remains lower than that of the general population. Whether this is because of incomplete restoration of normal valvular and myocardial function, or a result of complications that arise from prosthetic valves remains unknown. Nevertheless, the identification of prosthetic valve dysfunction remains a critical component in the management of such patients. Prosthetic dysfunction and complications are often recognized due to a change in the clinical status of the patient, but at times can be detected during routine screening TTE in the asymptomatic patient. Complications affecting prosthetic valves are vastly different depending on the timing of the complication after implantation. Early complications are typically related to technical related challenges of implantation of the valve, usually paravalvular leak in the setting of substantial annular calcium requiring debridement. These are usually mild in severity and may be medically managed in most situations. Other complications can include infectious endocarditis, and this has remained a periprocedural complication with high morbidity and mortality, even despite perioperative antibiotics [25, 26].

Long-term complications associated with prosthetic valves include thromboembolism, pannus ingrowth infective endocarditis, hemolytic anemia, prosthesis-patient mismatch (PPM), and of course complications secondary to anticoagulation. Some, such as thromboembolism, are far more common in mechanical valves. Others such as structural valve degeneration from tissue changes and degeneration, fibrosis, calcification, tearing, and perforation, on the other hand, are far more common in bioprostheses. Some of the older mechanical valves did exhibit some level of structural valve degeneration, such as strut fracture with disk embolization of the Bjork-Shiley valves and ball variance of the Starr-Edwards ball in cage prosthesis. However, modern mechanical valves are typically quite durable [27, 28]. Expected lifespans for mechanical valves exceeds 35 years for the SJM Regent and 50 years for the Starr Edwards valves [29, 30, 31, 32, 33]. Therefore degeneration of mechanical valves is not encountered routinely in clinical practice. This review will focus on complications of prosthetic valves that can be identified and managed with the use of echocardiography.

Prosthetic valve thrombosis can have catastrophic consequences to the patient; they are far more common in patients with mechanical valves compared to bioprosthetics, but can still present in the latter [34]. Clinical suspicion for prosthetic thrombosis should be raised by findings of heart failure, stroke, or change in auscultory findings of the valve, particularly in the setting of subtherapeutic or inadequate anticoagulation. Doppler echocardiography will demonstrate a reduced EOA, as well as increased peak and mean gradients. EOA can easily be calculated for the aortic position by using the continuity method: since flow will be equal through the LVOT and through the aortic valve, and since flow can be calculated by multiplying the time velocity integral (TVI) through the orifice and the surface area of the orifice, the EOA simply equals the (${\mathrm{TVI}}_{\text{LVOT}}$) (LVOT area)/(${\mathrm{TVI}}_{\text{AVR}}$). The EOA should be indexed (EOAi) to body surface area (BSA) as well as compared with gradients to ensure concordance between them. Different combinations of gradients with EOAi can assist with determination of the pathological state characterizing the valve. Additionally, the velocity profile for prosthetic thrombosis will also be distinct from one with other pathologies such as patient prosthesis mismatch or high flow states leading to elevated gradients. The continuity equation can also be used to estimate the EOA of a mitral valve as well.

**Fig. 2.**
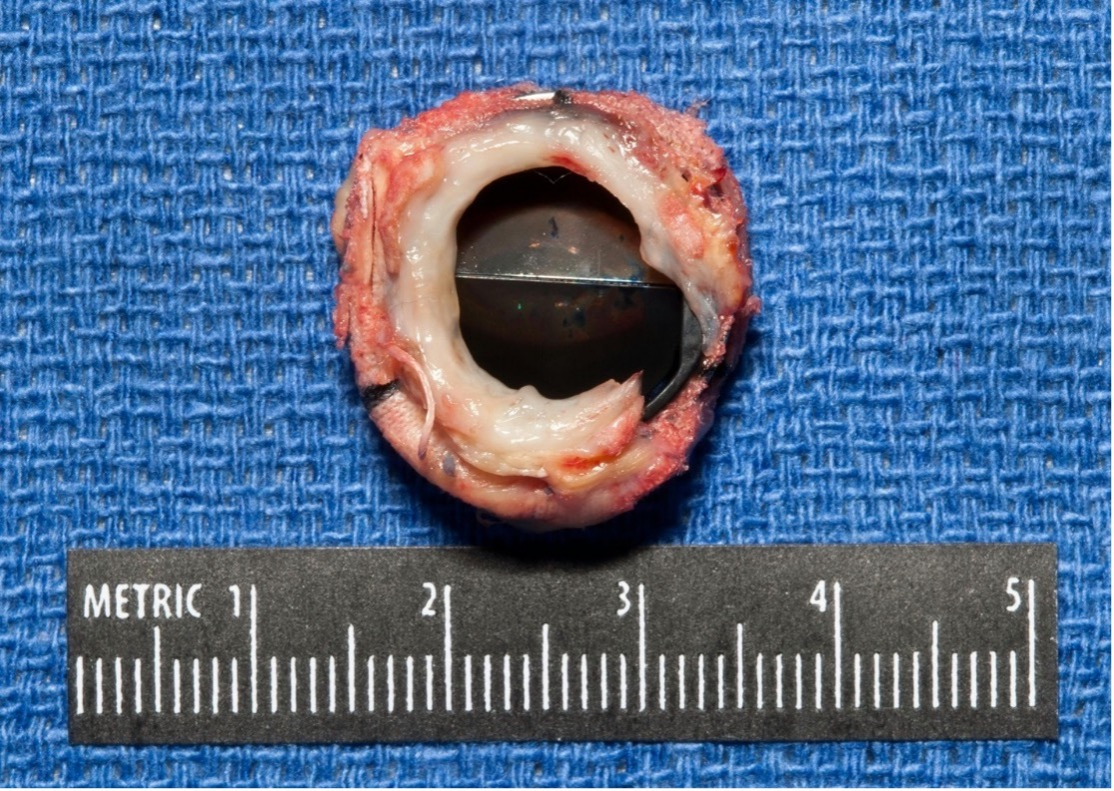
**An image of a prosthetic aortic valve with subvalvular pannus ingrowth leading to significant obstruction and stenosis after valve explant**. The orifice area of the surgical specimen showed excellent correlation with calculated orifice area via Doppler echocardiography.

Pannus ingrowth results from interaction between the prosthetic valve and host, which leads to fibrinous deposition on the valve (Fig. 2). This occurs with both bioprosthetics and mechanical valves, but is more common in the aortic position. Pannus ingrowth will eventually lead to obstructive hemodynamics similar to thrombus formation. As the clinical management of the two are entirely distinct, the diagnosis needs to be differentiated from valve thrombosis. Two-dimensional echocardiographic features are helpful in identifying the presence of pannus which will reveal a dense mass, though this may not always be visualized. Leaflet and occluders will have normal motion, and any abnormal motion should raise suspicion for prosthetic thrombosis instead of pannus.

Infective endocarditis (IE) in prosthetic valves is a serious complication, and is associated with mortality rates as high as 20–50%, though this rate has decreased over time [35, 36]. Prosthetic valves are associated with a higher risk of IE than native valves as well, and this remains true for both mechanical and bioprosthetic valves [37]. There is a broad spectrum of symptoms with which patients can present with, which can lead to misdiagnosis. Diagnosis is made by using the Modified Duke Criteria, in which echocardiography, along with positive blood cultures, is considered a major criteria. In the case of prosthetic valves, particularly mechanical prostheses, TEE is essential to ensure adequate visualization of all aspects of the prosthesis despite shadowing. Imaging should be done carefully to assess for the presence and size of the vegetation, the structural integrity of the valve and its competence, and perivalvular extension of infection such as abscesses and fistulae. These complications may be present even in a case where vegetation is not clearly manifest. The extent of the infection should also be carefully assessed, since infection may spread from the initial valve to involve other native or prosthetic valves.

**Fig. 3.**
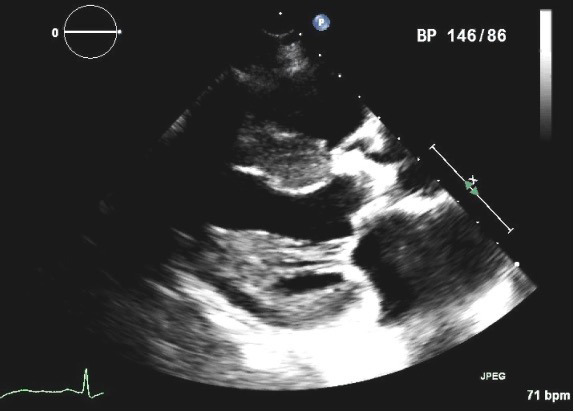
**Parasternal long axis view of a four year old 21 mm St Jude Trifecta pericardial aortic valve in a patient who presented with progressive exertional dyspnea and presyncope and was found to have severe prosthetic obstruction**. Note the highly echogenic aortic bioprosthesis, suggesting a heavily calcified valve with stenotic orifice, which was confirmed during valve replacement.

Structural valve deterioration (SVD) occurs almost exclusively in bioprosthetic valves, and is due to a combination of leaflet calcification and disruption of the collagen fibers composing of the valve (Fig. 3). These lead to progressive stiffening resulting in stenosis of the prosthesis, or tearing of the leaflets with the expected regurgitation that ensues. A less common form of SVD is stent creep which occurred more frequently in older generation bioprosthetics. This was characterized by an inward deflection of the stents and resulted in stenosis.

Patient prosthesis mismatch is a state in which a normally functioning prosthetic valve is implanted in a patient such that its EOA is too small with respect to the patient’s body size. PPM results in elevated transvalvular gradients, and has been associated with a host of adverse clinical outcomes. This includes reduced LV mass regression after implant, reduced LV systolic function, decreased improvement in functional status, and increased mortality both in the early post-surgical period and during long-term follow-up [38, 39, 40, 41, 42, 43]. There are some studies, however, that have failed to demonstrate an association of PPM with increased mortality with small amounts of PPM in both aortic and mitral positions [44, 45, 46]. Regardless, selection of an appropriately sized prosthesis, particularly for those with recued LV systolic function, is of paramount importance during the planning stages of prosthetic valve implant. The question of how precisely to define PPM is a difficult one. Certainly, Doppler echocardiography will demonstrate elevated gradients with a smaller than expected EOA, but the contour of the CW Doppler jet will be normal, rather than demonstrate the rounded symmetric morphology that would be characteristic of an obstruction. The indexed EOAi has consistently been found to correlate with postoperative gradients, and depending on the location of the prosthesis as well as the precise EOAi, the PPM may be characterized as mild, moderate, or severe. An EOAi <0.85 ${\mathrm{cm}}^{2}$/$m^{2}$ (severe <0.65 ${\mathrm{cm}}^{2}$/$m^{2}$) for aortic prosthesis and an EOAi <1.2 ${\mathrm{cm}}^{2}$/$m^{2}$ (severe <0.9 ${\mathrm{cm}}^{2}$/$m^{2}$) are the commonly accepted cutoffs for PPM. The EOAi may be underestimated in obese patients with a body mass index of greater than 30 kg/$m^{2}$, and so lower cutoffs if <0.70 ${\mathrm{cm}}^{2}$/$m^{2}$and <1.0 ${\mathrm{cm}}^{2}$/$m^{2}$for aortic and mitral positions respectively are recommended instead [47].

Paravalvular leak (PVL) occurs between the interface of the annulus of the native valve and the prosthetic sewing ring. PVL occurs due to suboptimal surgical implantation, infection, suture dehiscence, or extensive calcification of the annulus. It is more common in patients with percutaneously implanted prostheses compared to those surgically implanted. Trivial or mild PVL that bear no hemodynamic consequences are managed by observation, but larger orifices leading to more severe PVL may lead to substantial and clinically significant amounts of fragmentation hemolysis. Additionally, high output heart failure may ensue, and surgical or percutaneous closure of the PVL may become indicated in such a case [48, 49]. Color Doppler is the mainstay in diagnosing the presence and magnitude of PVL, and TEE may be necessary to differentiate it from intravalvular prosthetic leaks. PVL jets may be single or multiple, and usually are eccentric. A thorough and methodical approach should be undertaken by the echocardiographer to ensure adequate interrogation at multiple angles, and to ensure that the etiology and hemodynamic effects of the PVL are fully appreciated.

## 5. Echocardiographic Assessment: Aortic Valve Prostheses

The following sections will highlight the unique echocardiographic features of prosthetic valves beyond those of the general features that have already been described.

The normal hemodynamic profile of an aortic prosthesis mimics that of mild native aortic stenosis, and therefore maximal velocity will typically be >2 m/s. The contour of the Doppler profile will be triangular in shape, with rapid acceleration and peaking of velocity during early systole. A peak velocity >3 m/s should raise suspicion for a pathological state. Parameters such as the acceleration time and the ratio of ${\mathrm{TVI}}_{\text{LVOT}}$/${\mathrm{TVI}}_{\text{AVR}}$, known as the dimensionless index (DVI), can be used to determine whether a pathological state or an improper interrogation exists. The clinical presentation of the patient, such as a new murmur or congestive heart failure should always prompt a high suspicion for true prosthetic dysfunction.

**Fig. 4.**
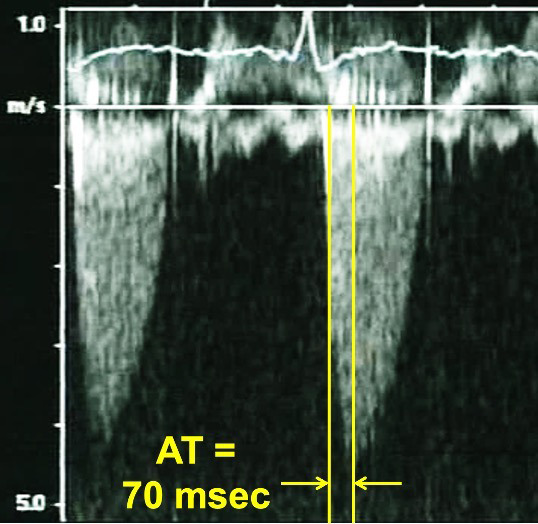
**Continuous Wave Doppler of a normally functioning aortic prosthesis with functionally obstructive hemodynamics**. Note the triangular contour of the Doppler jet with a rapid acceleration time (AT) of 70 msec.

**Fig. 5.**
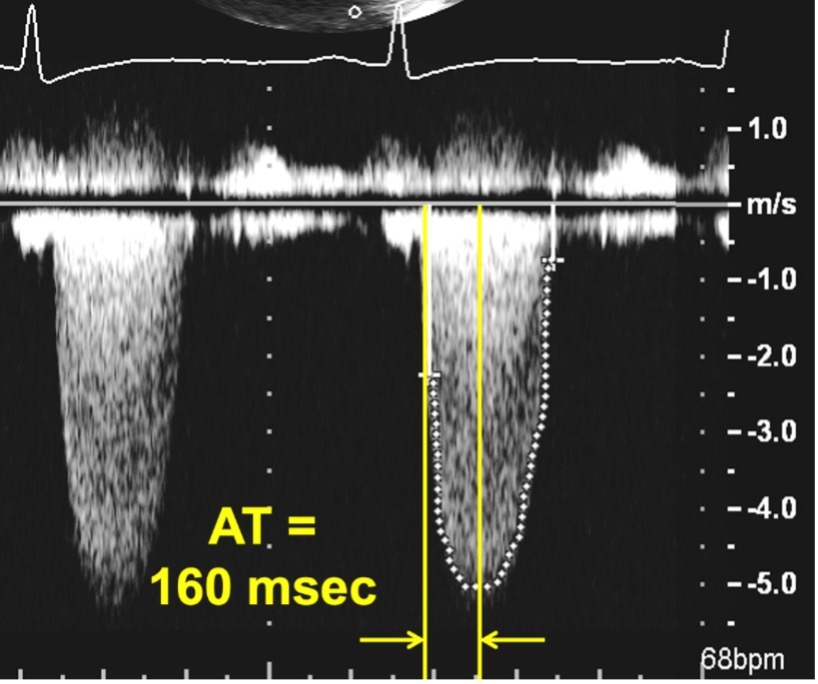
**Continuous Wave Doppler of an aortic prosthesis with structurally obstructive hemodynamics**. Note the rounded contour of the Doppler jet, with an acceleration time (AT) of 160 msec.

Progressive stenosis will lead to a prolonged acceleration time (AT), which is the time to the peak of the jet velocity as a result of delayed peaking of the velocity during systole. Therefore, the Doppler profile contour of an aortic prosthesis with stenosis with thrombi or pannus formation will be blunted and rounded as opposed to the triangular shape characteristic of a normally functioning prothesis (Figs. 4,5). This can be quantified by the ratio of the AT to the total ejection time (ET) over which blood flow occurs during systole, as a normal AT/ET ratio is less than 0.32. The AT as well as the AT/ET can also help distinguish true prosthetic obstruction from other conditions that confer a “functional” obstruction such due to high flow states (which can result from anemia, thyrotoxicosis, AV fistulas, or significant aortic regurgitation), pressure recovery, or patient prosthesis mismatch that can also lead to an elevated mean aortic prosthetic gradient. A functional obstruction will present with a peak velocity greater than 3 m/s, but the AT will be less than 80 ms, and the AT/ET, though it may be mildly elevated will not typically be greater than 0.37 [50, 51].

The DVI can provide incremental information to the AT. The DVI is a ratio of the TVI of the LVOT to that of the aortic prosthesis (DVI = ${\mathrm{TVI}}_{\text{LVOT}}$/${\mathrm{TVI}}_{\text{AVR}}$). It can also be estimated as the ratio of the velocities (rather than the TVIs) of the LVOT and aortic prosthesis. This is based on the assumption that the contours of the TVI of the LVOT and aortic prosthesis are the same, and without which the estimation will introduce error and therefore should not be used. The DVI also eliminates the LVOT dimension, which is a potential source of error that is included in the continuity equation. The DVI is a good initial measurement to screen for significant valve obstruction, as a DVI <0.25 is highly suggestive of this. In a cohort of patients with severe aortic stenosis of St Jude Medical prostheses, the mean DVI was 0.19 ± 0.05, whereas a matched control with normal prosthetic function was 0.39 [52, 53]. The DVI is additionally not affected by high flow conditions, as the flow through the aortic prosthesis is proportionally increased to the increased flow through the LVOT, and the ratio remains the same (Table 1) [53, 54].

**Table 1.** **Doppler parameters of prosthetic aortic valve function**.

	Acceleration time (ms)	Dimensionless index	Acceleration time/ejection time	Effective orifice area index (${\mathrm{cm}}^{2}$/$m^{2}$)
Normal	<80	>0.30	<0.32	>1.2
Possible stenosis	80–100	0.25–0.29	>0.37	0.8–1.2
Severe stenosis	>100 ms	<0.25	>0.4	<0.8
High flow state	<100 ms	0.25–0.29	<0.37	>1.2
Patient prosthesis mismatch	<100 ms	0.25–0.29	<0.37	≤0.65 severe
				0.66–0.84 moderate [54]

**Fig. 6.**
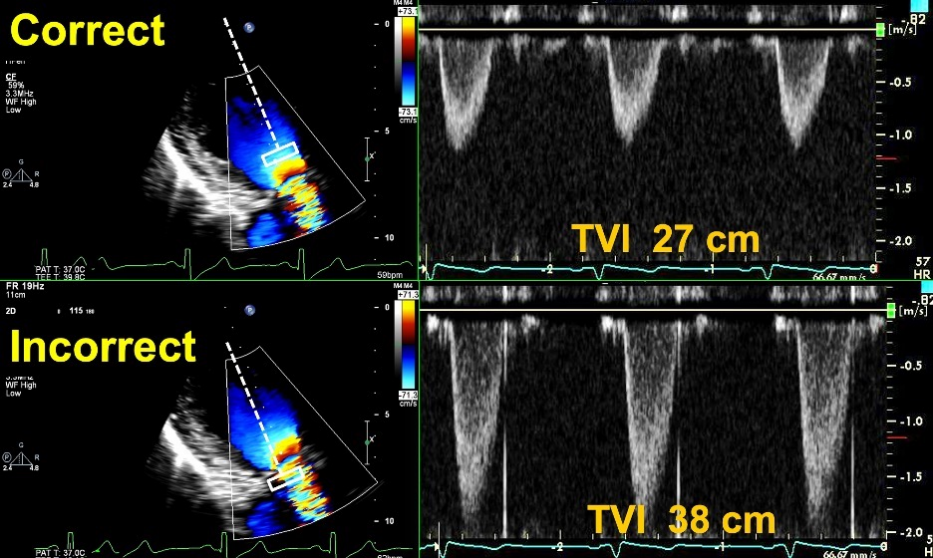
**Correct and Incorrect method of measuring the TVI of the LVOT. ***Top Images*: Pulse Wave Doppler with sample volume placed immediately proximal to the zone of flow acceleration within the LVOT, with an accurate TVI of 27 cm. *Bottom images*: Pulse Wave Doppler with sample volume placed within the zone of flow acceleration leading to a spuriously elevated TVI of 38 cm. This can lead to an elevated DVI in a patient with an obstructive prosthesis, and care should be taken to avoid this error.

As always, these parameters should not be interpreted in isolation because of the significant variability and overlap in the values due to various valve types and sizes. Rather they should be interpreted in conjunction with one another, and within the context of the patient’s clinical presentation. If there are discordant values, an explanation for the discordance should be sought. With a normal DVI >0.30 but a rounded contour with an elevated AT, it is likely that the DVI is spurious rather than the AT, and there is improper interrogation of the PW Doppler with placement of the sample volume within the zone of flow acceleration in the LVOT leading to an artificially increased ${\mathrm{TVI}}_{\text{LVOT}}$ [55] (Fig. 6). This is true also if a patient presents with a DVI <0.25, but with an AT <100 ms, where the AT should take preference over the DVI, as the ${\mathrm{TVI}}_{\text{LVOT}}$ may once again be spuriously lower that its true value due to a sample volume too apical from the prosthesis. By analysis of the DVI, the CW Doppler velocity profile of the aortic prosthesis, and the EOAi, a variety of conditions associated with an increased mean pressure gradient across an aortic prosthesis can be delineated.

Prosthetic valve regurgitation is notably not as well-described in the literature as compared to prosthetic stenosis or native aortic regurgitation (AR). Additionally, the assessment of its severity is more challenging because of a high prevalence of eccentric jets or paravalvular regurgitation. Color Doppler plays a large role in determining the mechanism of the AR as well as quantification of its severity, which is quite similar to that of native AR. Parameters such as the ratio of the jet diameter/LVOT diameter can be used, though these should be applied primarily to central jets. Additionally, the width of the vena contracta may be difficult to assess in the long-axis. Spectral Doppler is primarily used to determine pressure half-time (PHT), which if <200 ms suggests severe AR, and if >500 ms suggests mild AR. Intermediate values are less specific, as with native AR [56]. These should be used in caution with patients suspected of acute prosthetic AR, however.

Paravalvular leaks or regurgitation (PVL) should be distinguished from intravalvular regurgitation. This typically is a result of disruption of the sewing ring suture, and usually ensues from infectious endocarditis and abscess formation. Color Doppler with TTE and TEE may be useful in assessing the location of the jet, and thereby identifying the regurgitant jet as paravlvular. Three-dimensional echo with color may be particularly helpful in this situation. The measurement of the ration of the sewing ring circumference to the length of suture dehiscence may assist in the assessment of the size of the PVL. A ratio <10% is suggestive of a mild PVL, whereas >20% is severe [57].

## 6. Echocardiographic Assessment of Mitral Valve Prostheses

Though the mitral valve can be imaged with TTE using a number of available windows, acoustic shadowing often limits optimal visualization particularly with mechanical valves. Therefore, a complete examination of a mitral prosthesis often involves both TTE and TEE views when there is high threshold of suspicion for dysfunction.

Echocardiographic parameters that should be measured during a complete mitral prosthesis evaluation include the peak mitral inflow velocity, the mean pressure gradient, pressure half time (PHT), DVI, and EOA (Table 2). Because transmitral velocities and gradients are dependent on heart rate (HR), the HR should be noted on every report to contextualize the hemodynamic findings. Left atrial, left ventricular, and right ventricular enlargement or dysfunction or an elevated pulmonary artery systolic pressure can also hint at underlying mitral prosthetic dysfunction.

**Table 2.** **Doppler parameters to grade mitral prosthetic obstruction**.

	Normal	Possible obstruction	Significant obstruction
Pressure half time (ms)	<130	130–200	>200
Peak velocity (m/s)	<1.9	1.9–2.5	≥2.5
Mean gradient (mmHg)	≤5	6–10	≥10
Effective orifice area (${\mathrm{cm}}^{2}$)	≥2	1–2	<1
Dimensionless Index (DVI)	<2.2	2.2–2.5	>2.5
Doppler Parameters to Grade Mitral Prosthetic Regurgitation			
	Mild	Moderate	Severe
Vena contracta (mm)	<3	3–5.9	≥6
Regurgitant jet area (${\mathrm{cm}}^{2}$)	<4	4–7.9	≥8
Dimensionless Index (DVI)	<2.2	2.2–2.5	>2.5

The peak mitral inflow velocity (E-velocity) can be used as an initial starting point in the assessment of mitral prostheses. If the peak velocity is <1.9 m/s, the prosthesis can be assumed to be normal in most patients barring severely depressed LV systolic function [58]. A normal E-velocity for a bileaflet mechanical mitral prosthesis can be up to 2.4 m/s, so there is some overlap in normal function, primary prosthetic dysfunction, and high flow states [59, 60]. Two-dimensional imaging to detect the presence of leaflet motion and the presence or absence of vegetation or thrombus can help differentiate these conditions.

**Fig. 7.**
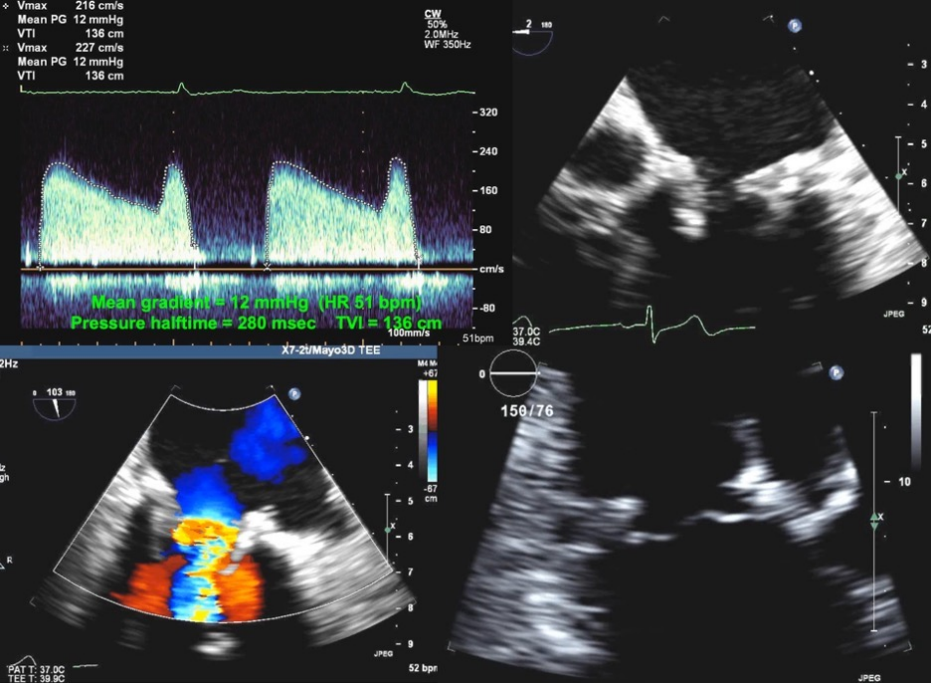
**Echocardiographic imaging of a patient with a mitral bioprosthesis and heart failure symptoms found to have prosthetic thrombosis. ***Top Left*: Continuous Wave Doppler in a patient with a 31 mm Hancock porcine mitral bioprosthesis who presented with dyspnea on exertion and lightheadedness. The mean gradient was 12 mmHg with a heart rate of 51 and a pressure halftime of 280 msec. The DVI was 5.7, with an EOAi of 0.44 ${\mathrm{cm}}^{2}$/$m^{2}$ all consistent with severe obstruction. *Top Right*: This is confirmed on transesophageal echo which shows an echogenic mass on the anterior leaflet that is highly mobile and independent of the prosthetic leaflets; additionally there is increased leaflet thickness and mobility on the live images. *Bottom Left*: Color Doppler demonstrates turbulence during diastole with transmitral flow due to the obstruction. Because of suspicion for mitral prosthetic thrombosis, the patient was initiated on warfarin therapy. *Bottom Right*: Transesophageal echo image of the mitral valve after 6 weeks of warfarin therapy showing complete resolution of the thrombosis. This case highlights the importance of recognizing bioprosthetic valve thrombosis on one’s differential with an incidence of 0.64% in the mitral position.

The mean gradient is normally <5–6 mmHg, though this depends on the type of the prosthesis [61]. Normally functioning cage-in-ball prostheses will of course have higher gradients, which can be as high as 10–12 mmHg [62]. Additionally, the PHT of a normally functioning mitral prosthesis will rarely exceed 130 msec. Conversely, a prolonged measurement >200 msec suggests significant obstruction (Fig. 7). However, myocardial compliance, relaxation and loading conditions can greatly affect the PHT and this value should not be taken in isolation. It should be noted that calculation of EOA using PHT as is done commonly with native mitral valves is not appropriate because of the assumptions inherent in the equation that are not valid with mitral prostheses. EOA should be calculated using the continuity equation. Even with this method, the EOA will be more accurate for bioprosthetics and single tilting disc mechanical valves where there is a single orifice as compared to bileaflet mechanical prostheses. This is because the smaller central orifice will have a higher velocity the other larger orifices, and the TVI of the prosthetic valve will be overestimated, leading to an underestimation of the EOA [61]. Typically an EOA of <1 ${\mathrm{cm}}^{2}$ suggests severe stenosis of the prosthesis.

**Fig. 8.**
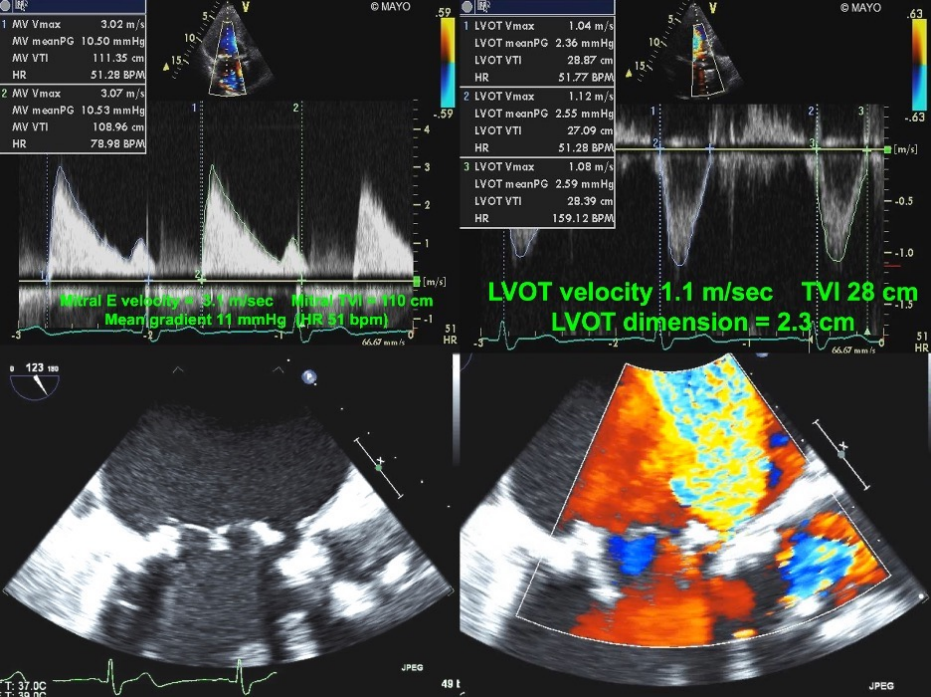
**Echocardiographic imaging of a patient with a mitral bioprosthesis and prosthetic dehiscence**. *Top Left and Right*: Continuous Wave Doppler of a male with a Biocor porcine mitral bioprosthesis just 5 months previously. The mean gradient was 11 mmHg with a heart rate of 51. The DVI was 4.0, with an EOAi of 0.48 ${\mathrm{cm}}^{2}$/$m^{2}$ which may mislead towards a diagnosis of obstruction. However, the pressure half time was 120 msec, which is too short to be seen in severe structural obstruction. *Bottom Left*: Transesophageal 2-D image showed rocking of the mitral prosthesis with evidence of a paravalvular gap. *Bottom Right*: Color Doppler shows torrential MR secondary to a paravalvular leak with an eccentric jet. This was confirmed as prosthetic dehiscence during surgical correction.

Prosthetic mitral regurgitation (MR) is often rendered challenging with TTE because of acoustic shadowing, and TEE is frequently required for optimal characterization. Determination of the severity of prosthetic MR is similar to that of native valves, with an elevated E velocity and a dense CW regurgitant jet (Fig. 8). The regurgitant jet area may be used with caution, as it may reflect severity if it is central in origin. A large and wide jet with ≥8 ${\mathrm{cm}}^{2}$ reflects significant MR [63]. Additionally, vena contracta of ≥6 mm reflects a large regurgitant volume. There also may be incremental benefit in the form of superior delineation of the precise location, shape, and severity of prosthetic MR paravalvular leaks with the use of three-dimensional (3D) echocardiography [64].

**Fig. 9.**
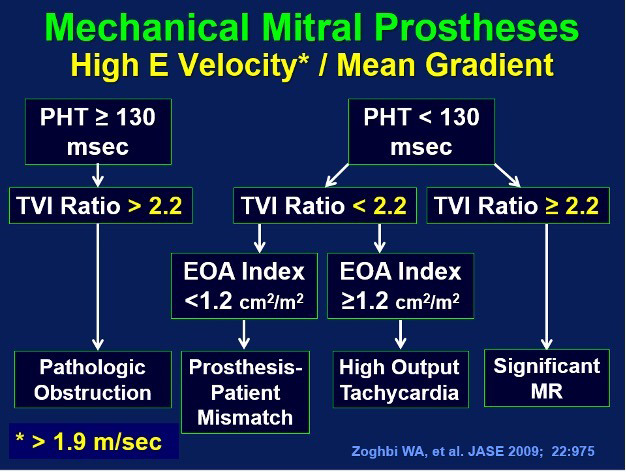
**A mitral pressure half time of ≥130 msec associated with a ${\mathrm{TVI}}_{\text{𝐌𝐕𝐑}}$/${\mathrm{TVI}}_{\text{𝐋𝐕𝐎𝐓}}$ ratio of >2.2 for mechanical mitral prostheses with an increased mean pressure gradient is consistent with structural obstruction such as with thrombosis, pannus ingrowth, or occluder disc immobility**. With a ${\mathrm{TVI}}_{\text{MVR}}$/${\mathrm{TVI}}_{\text{LVOT}}$ ratio of >2.2 with a PHT of <130 msec, functional stenosis due to mitral regurgitant inflow volume (reflected by increased ${\mathrm{TVI}}_{\text{MVR}}$) crossing the mitral prosthesis is likely. In high cardiac output states or with tachycardia, the flows across the mitral prosthesis and LVOT would both be increased as reflected by a TVI ratio of <2.2 and a mitral EOAi of ≥1.2 ${\mathrm{cm}}^{2}$/$m^{2}$. Mitral prosthesis patient mismatch is likely if the EOAi is <1.2 ${\mathrm{cm}}^{2}$/$m^{2}$ with a PHT of <130 msec and a ${\mathrm{TVI}}_{\text{MVR}}$/${\mathrm{TVI}}_{\text{LVOT}}$ ratio <2.2. Modified and adapted after reference [22].

The DVI for mitral prostheses may be confusing as the ratio is ${\mathrm{TVI}}_{\text{MVR}}$/${\mathrm{TVI}}_{\text{LVOT}}$ where the ${\mathrm{TVI}}_{\text{LVOT}}$ is present in the denominator and not the numerator. Thus, a smaller TVI ratio is normal, and larger values suggest obstructive pathology if the PHT is >130 msec, or increased flow (functional stenosis) if the PHT is <130 msec. This is born out in studies which identify a DVI <2.2 as normal, and higher values abnormal with a very good positive predictive value. [59]. Using the mitral PHT and the ${\mathrm{TVI}}_{\text{MVR}}$/${\mathrm{TVI}}_{\text{LVOT}}$ ratio of either >2.2 or <2.2, a variety of conditions causing an increased mean pressure gradient across the mechanical mitral valve prostheses can be delineated as shown in the algorithm in Fig. 9. The same algorithm and be applied to bioprosthetic valves, but with a ${\mathrm{TVI}}_{\text{MVR}}$/${\mathrm{TVI}}_{\text{LVOT}}$ ratio cut point value of 2.3 for pericardial mitral bioprostheses and 2.6 for porcine mitral bioprosthesis.

## 7. Echocardiographic Assessment of Tricuspid and Pulmonary Valve Prostheses

There is a dearth of literature on pulmonary and tricuspid prostheses, and the little data that there is comes mainly from pediatric studies in patients with congenital heart disease. The pulmonary valve in particular is challenging to assess by both TTE and TEE, because of its anterior and superior location [65]. With TTE, the pulmonary valve is best imaged from the parasternal short axis at the aortic valve level, as well as with the right ventricular outflow tract view and subcostal view. A cranial tilt gives a more clear view of the pulmonary valve and artery.

Despite the paucity of data regarding identification and quantification of pulmonary prosthetic dysfunction, some considerations should be taken into account. In general, a peak velocity of >3.2 ms (mean gradient ≥20 mmHg) for bioprosthetic or >2.5 m/s (mean gradient ≥15 mmHg) for homografts should raise suspicion for obstruction [66, 67, 68]. In addition to valve gradients and velocities, right ventricular (RV) systolic hypertension and RV systolic dysfunction can be a marker for prosthetic pulmonary stenosis. Direct 2-D imaging revealing leaflet or cusp thickening or immobility, as well as color Doppler with turbulence and narrowing of the color map through the valve is also suggestive of prosthetic stenosis.

Prosthetic pulmonary regurgitation (PR) is diagnosed with color Doppler revealing diastolic flow in the RVOT directed towards the right ventricle. Significant PR is characterized by the duration of the flow, as more severe PR will have flow throughout diastole [69]. This will additionally lead to an intense spectral Doppler signal. Nevertheless, the duration of the flow can be misleading, as severe PR may lead to equalization of the RV pressure with diastolic pulmonary artery pressure, and thereby lead to a very short duration of flow [70]. Additional parameters that can be used to determine severity of prosthetic PR is the jet width: if it occupies >50–65% of the RVOT, this suggests severe PR, whereas a narrow jet <25% of the pulmonary annulus suggests mild PR [71]. This may be less reliable in the setting of an eccentric jet or a paravalvular leak which may underestimate the severity of the regurgitation.

An additional parameter that may be helpful in the determination of the severity of prosthetic PR is the PHT. As in MR, a short PHT, defined as <100 ms) is consistent with severe PR. In this case, a sine shaped wave with early termination of the flow would be seen [56]. A short PHT is not a specific marker for severe PR because it is dependent on other parameters, such as diastolic intrapulmonary pressures as well as the diastolic properties of the RV [72, 73]. As such, a restrictive RV may present with a short PHT without severe PR. Other quantitative variables that require calculations such as regurgitant volume and fraction can also be utilized, though they are subject to errors because of the difficulty in measuring the pulmonary annulus size. Additionally, they have not been validated as they have been with AR.

As with prosthetic pulmonary obstruction, significant prosthetic PR should be suspected if there are characteristic upstream effects on the RV. RV dilatation or diastolic flattening of the interventricular septum due to volume overload suggests severe PR. Though this is not specific, it does have a good negative predictive value, as a normal RV size does suggest the absence of chronic severe prosthetic PR.

Tricuspid prostheses, in contrast to prosthetic ones, are anterior in location, and therefore assessment is easier and in fact may be superior with TTE to that of TEE [74]. A standard examination of a tricuspid prosthesis should include a medially directed parasternal long axis of the RV inflow, a parasternal short axis view at the left of the aortic valve, an apical four-chamber view, and a subcostal view. Because tricuspid valve hemodynamics are influenced by respiration, several cardiac cycles should be averaged even if the patient is in sinus rhythm.

A normally functioning tricuspid prosthesis will have an inflow peak velocity of <1.9 m/s or a mean gradient <6 mmHg [75, 76]. However, there may be significant variation with transtricuspid gradients and velocities with varying respiration, heart rate, and RV loading conditions. Nevertheless in the absence of tachycardia, elevated velocities and gradients should raise suspicion for possible obstruction. The PHT can also help guide diagnosis, as obstructive pathology will prolong the PHT. In a group of 46 patients with St. Jude Medical tricuspid prostheses, the mean PHT was 123 ms, whereas those with obstruction had a PHT of 272 ms. None of the patients with obstruction had a PHT <160 ms [77].

Unlike flow gradients and PHT which are both dependent on flow and loading conditions, the DVI is less flow-dependent; a DVI ≥3.2 for biological tricuspid prostheses and ≥2 for mechanical prostheses should raise suspicion for tricuspid stenosis [74, 78]. Although EOA should also be independent of flow and loading conditions, cut-off values for EOA have not been validated for tricuspid prostheses.

The assessment and quantification of prosthetic tricuspid regurgitation (TR) is similarly lacking in robust data, and therefore standard methods used for MR and native TR are extrapolated to the prosthetic tricuspid population. Color Doppler is a primary screening tool to detect the presence of prosthetic TR. In general, a larger color jet that extends further into the RA suggests more severe TR than a smaller jet. However, this is highly subjective and also dependent on the direction of the jet as well technical factors of the Doppler settings. A more objective parameter is the vena contracta, which for native valves has a cutoff value ≥7 mm for severe TR, though this may be obscured by shadowing from the prosthesis [79, 80]. Similarly, PISA may be used to quantify regurgitant volume and fraction, as it is for native valves, though neither vena contracta nor PISA have been specifically validated for prosthetic TR.

Spectral Doppler parameters that can be measured include the tricuspid valve inflow peak velocity (E-velocity). As in MR, an elevated E-velocity (>2.1 cm/s) should raise suspicion for significant TR, if there is no evidence of tricuspid stenosis [81]. Holosystolic reversal of flow in the hepatic vein is specific for severe TR. Additionally assessment of the right heart for RA and RV enlargement and septal flattening with diastole as well as inferior vena cava for dilatation and respiratory variation can also assist with the identification of significant prosthetic TR.

## 8. Conclusions

Prosthetic valves are frequently encountered by cardiologists because of the increasing incidence of valvular heart disease in the general population. As such, clinicians need to be cognizant of the management of prosthetic valves.

Echocardiography is the foundational imaging modality for the screening of prosthetic valve function and diagnosis of prosthetic valve dysfunction. It provides both anatomic and functional information with a high degree of accuracy, reproducibility, and fidelity. Although prosthetic valve dysfunction may present with a multiplicity of echocardiographic findings, many are shared by native valves, and the fundamental principles for interpretation remain the same. Echocardiographic interpretation of prosthetic valves requires a thorough understanding of ultrasound physics, an understanding of the generalities and specifics of prosthetic hemodynamics, and knowledge of the specific pathologies that may arise and lead to prosthetic dysfunction. Meticulous attention to detail needs to be paid during the interpretation of these studies to ensure that subtle findings that may signal significant prosthetic dysfunction are not overlooked.

The general approach includes a review of prior images and serial comparison of 2-D images as well as color and spectral doppler to assess hemodynamic function of the prosthetic. A methodical, comprehensive, and consistent approach to the echocardiographic interpretation of prostheses will ensure that all salient features and aspects are assessed.
